# Understanding Treatment Completion and Tolerability of Intravesical Bacillus Calmette-Guérin (BCG) for Non-muscle Invasive Bladder Cancer in a Single UK Health Board

**DOI:** 10.7759/cureus.98655

**Published:** 2025-12-07

**Authors:** Sami Mohammed, Michael Aniah, Chalint Saougki, Ahmed E Ibrahim, Katie E Badawy, Caitlin Mitchell, Badreldin Mohamed, Shaza Faycal Mirghani, Abdolazeem Elnour, David H Lau

**Affiliations:** 1 Urology, Glangwili General Hospital, Carmarthen, GBR; 2 Urology, South Tyneside Hospital, South Shields, GBR; 3 General Surgery, Glangwili General Hospital, Carmarthen, GBR; 4 Vascular Surgery/General Surgery, Cumberland Infirmary, Carlisle, GBR; 5 General Surgery, Barnet General Hospital, London, GBR; 6 General Surgery, Sandwell and West Birmingham NHS Trust, Birmingham, GBR; 7 General Surgery, Withybush General Hospital, Haverfordwest, GBR

**Keywords:** bcg induction, intravesical bcg therapy, non-muscle-invasive bladder cancer(nmibc), recurrence following bcg therapy, systemic toxicity of bcg

## Abstract

Background

Intravesical Bacillus Calmette-Guérin (BCG) is the standard immunotherapy for high-risk non-muscle invasive bladder cancer (NMIBC). A considerable proportion of patients do not respond adequately to this treatment. This study aims to analyze the rates of and reasons for discontinuation of BCG therapy within a single UK health board.

Methods

A retrospective study of 40 patients who underwent BCG treatment for NMIBC at Glangwili General Hospital (Hywel Dda University Health Board) from July 2021 to July 2025. Data on patient demographics, tumor characteristics, treatment adherence, and adverse events were collected from medical records.

Results

Of the 40 patients, 60.0% (n=24) did not complete the full BCG course. Only seven patients (17.5%) exhibited BCG intolerance, grade 2+ toxicities, or related admissions. Smoking status was poorly documented, available for only 10 patients (25.0%). Among these, 15% (n=6) were ex-smokers, 5% (n=2) were current smokers, and 5% (n=2) had never smoked. Adherence to dosing intervals was generally good; 16 of 24 non-completers (66.7%) and 10 of 14 completers (71.4%) received their doses on time (≤14 days). Tumor characteristics showed a high prevalence of high-grade tumors in both non-completers (13/16, 81.3%) and completers (8/10, 80.0%).

Conclusion

Within our Health Board, BCG failure is multifactorial. It is important to improve data collection, particularly on smoking status and reasons for discontinuation. This will provide us with a more comprehensive understanding of BCG failure and help us improve treatment protocols. A multidisciplinary approach is needed to optimize BCG adherence and effectively manage high-risk NMIBC.

## Introduction

Intravesical Bacillus Calmette Guérin (BCG) is an immunotherapy that is used as the treatment of choice in high-risk non-muscle invasive bladder cancer (NMIBC) after transurethral resection of the bladder tumor (TURBT), which helps in tumor regression and prevents recurrence [1]. Bladder cancer is the sixth most common cancer in the world, and NMIBC accounts for nearly 75% of bladder cancer [1]. High-risk NMIBC patients need one to three years of a full course of intravesical BCG as adjuvant treatment; if it fails, then radical cystectomy is necessitated [2].

BCG failure could be correlated with multiple factors, either patient-related or demographic-related factors, such as age, gender (Male or Female), and prognostic risk factors, such as smoking and some industrial substances, such as polycyclic aromatic hydrocarbons, or tumor related factors as size, number of tumours, degree of grading, +/- carcinoma in situ (CIS) and histological differentiation as Ta and T1 [2].

BCG failure is categorized into four groups. BCG-refractory tumor is defined as the presence of CIS without a papillary tumor after three to six months of re-induction or after the first maintenance in primary T1 high-grade cancer. BCG relapse occurs when the tumor recurs after a complete response to a full course of BCG. BCG-unresponsive tumor refers to a high-grade T1 or Ta tumor that reappears within six months of adequate induction and maintenance therapy, or carcinoma in situ that appears within 12 months of adequate BCG therapy, or high-grade disease that persists after an induction course alone. BCG intolerance occurs when side effects prevent adequate completion of BCG treatment [3].

Grades refer to the severity of adverse events (AEs) and are classified from 1 to 5. Grade 1 is mild symptoms that are mostly asymptomatic; only clinical observations are noted, and no intervention is necessary. Grade 2+ toxicities signify moderate side effects that are bothersome and interfere with daily activities, but are not immediately dangerous. They often require minimal, local, or non-invasive medical intervention. Grade 3 is severe or significant symptoms that require medical attention but are not immediately life-threatening, may necessitate hospitalization or an extended hospital stay, and can interfere with daily self-care activities. Grade 4 is life-threatening conditions that require urgent medical intervention. Grade 5 is resulting in death due to the adverse event. This classification helps in assessing and managing the impact of treatment-related side effects [4].

The primary objective of this study was to evaluate the reasons for discontinuation and failure of intravesical BCG therapy among high-risk NMIBC patients within the Health Board, and to identify factors associated with incomplete treatment. This retrospective design was chosen to evaluate real-world adherence to BCG therapy and identify practical barriers to completion, as these factors directly influence recurrence risk and guide future strategies for improving outcomes in high-risk NMIBC patients.

## Materials and methods

This retrospective observational cohort study was conducted at Glangwili General Hospital, part of Hywel Dda University Health Board, Wales, UK. All patients who received intravesical BCG for histologically confirmed high-risk NMIBC (Ta, T1, ±CIS) according to European Association of Urology (EAU) guidelines between July 2021 and July 2025 were included (n=40). Patients with muscle-invasive disease or those receiving mitomycin immunotherapy were excluded.

Data collected from the Welsh Clinical Portal electronic health records, operation notes, urology multidisciplinary team meeting records, clinic letters, and cystoscopy reports. Variables collected included patient demographics (age, sex, and smoking status when documented), comorbidities, tumour characteristics (stage, grade, size, multiplicity, and presence of concomitant carcinoma in situ), treatment details (induction and maintenance schedule, dose delays, and any modifications), adverse events (type, grade, management, and any hospital admission related to BCG therapy), and clinical outcomes (tumour recurrence, progression to muscle-invasive disease, cystectomy, and survival).

The primary outcome was BCG completion rate and reasons for discontinuation. Secondary outcomes included factors associated with failure and protocol adherence (dose interval ≤14 days). The study was registered as a service evaluation using fully anonymised retrospective data; no patient-identifiable information was extracted, and formal ethical approval was not required.

Data were analyzed descriptively using IBM SPSS version 21 (IBM Corp., Armonk, NY, USA). Results are presented as frequencies and percentages or means ± SD/medians (IQR) as appropriate. All patients meeting the inclusion criteria were analyzed regardless of missing variables. Missing data were neither imputed nor used for subgroup exclusion; comparisons were descriptive only.

## Results

**Figure 1:**
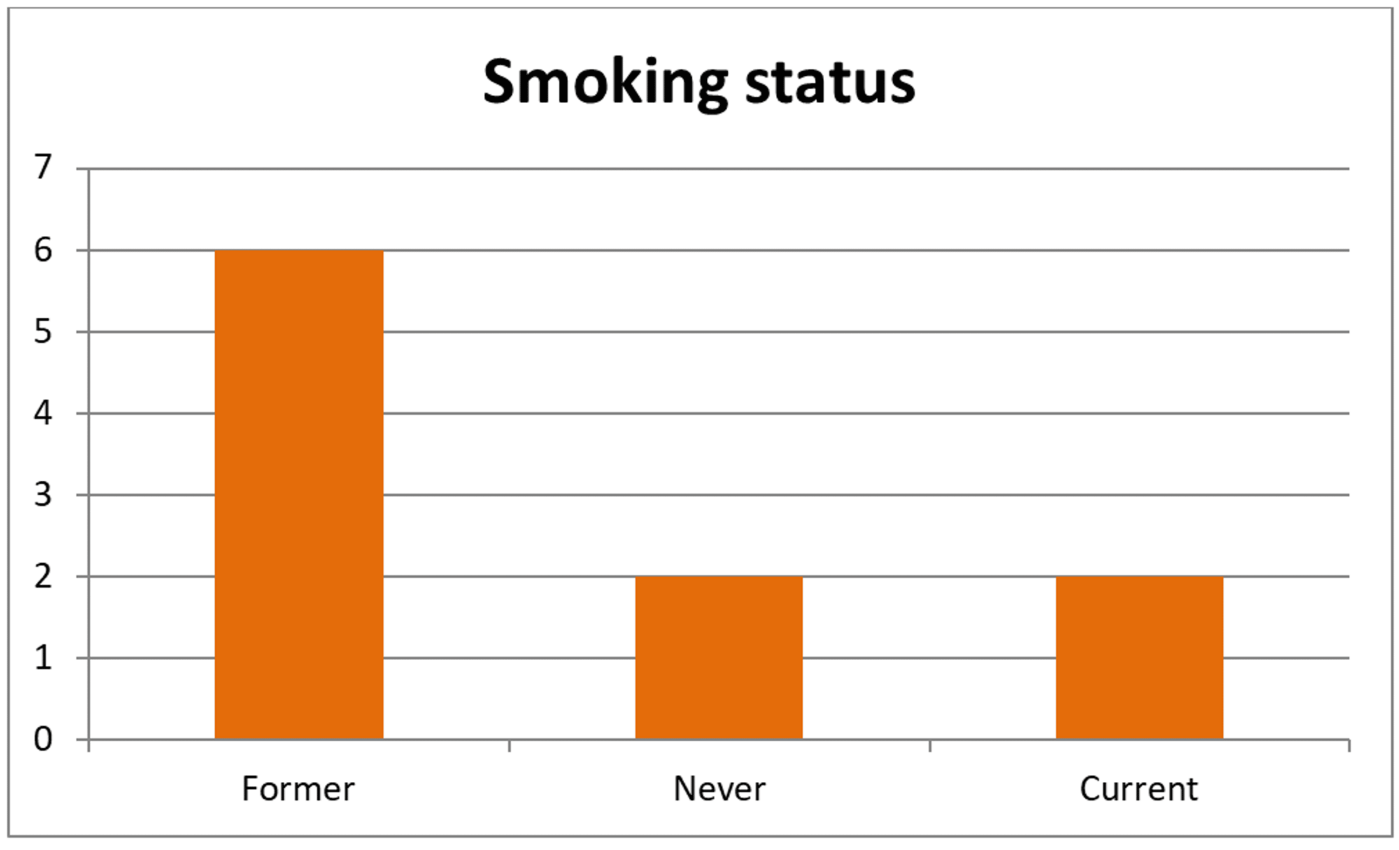
Documented smoking status in patients with NMIBC Former: Ex-Smokers Never: Non-Smokers Current: Current smokers NMIBC: Non-Muscle Invasive Bladder Cancer

The study group evaluated during the specified period consisted of 40 patients who received intravesical BCG treatment; 60% (n=24) did not complete the BCG therapy. The smoking status was poorly documented, available for only 10 patients (25.0%). Among 10 patients with documented smoking status, 60% were ex-smokers (n=6), 20% were current smokers (n=2), and 20% had never smoked (n=2). The documented smoking status in patients with NMIBC is shown in Figure 1.

**Figure 2:**
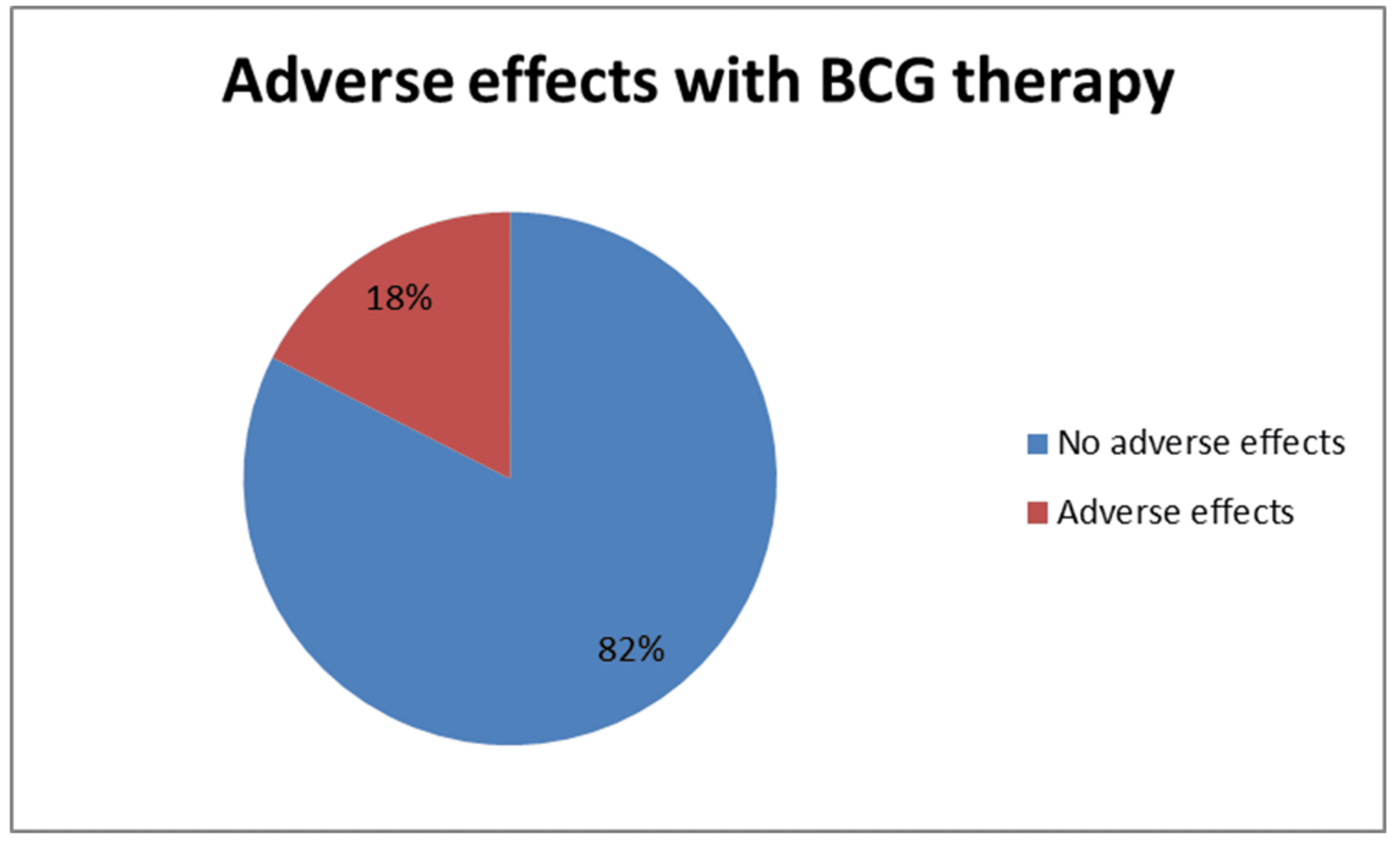
Percentage of patients experiencing adverse effects associated with BCG therapy BCG: Bacillus Calmette Guérin

Only 18% (n=7) of patients experienced grade ≥2 toxicity, intolerance, or BCG-related admission, as demonstrated in Figure 2. 

Sixty percent (n=24) did not complete the full course. Among the 24 patients who did not complete BCG therapy, seven discontinued due to intolerance or grade ≥2 toxicities. The remaining 17 cases were attributed to logistical challenges as scheduling delays, patient choice, or undocumented reasons, which could not be fully verified due to retrospective data limitations. Clinically relevant details of toxicity and intolerance among patients who discontinued BCG therapy are summarized in Table 1.

**Table 1:** Patients who experienced toxicities, intolerance, and BCG-related admissions. BCG: Bacillus of Calmette-Guerin (BCG) TB: Tuberculosis

Sex	Toxicities	Intolerance description	BCG related Admissions
M	Yes	Unwell after first dose, suspected TB	Yes
M	No	Can’t retain BCG initially, helped with pacey cuff, arthritis flare	No
M	No	Retention hard at start of administration of BCG, used catheter help	No
M	No	Not documented	No
F	No	Not documented	No
F	Yes	"Too draining" as patient described, stopped all instillations	No
M	Yes	"Too much side effects" patient described after three doses	No

Adherence to dosing intervals was generally good; 66% (n=16) of patients who did not complete BCG treatment received their therapy within a two-week interval between doses, and 72% (n=10) of completers received doses on time (≤14 days). Adherence to the protocol regarding dose timing is detailed in Table 2.

**Table 2:** Adherence to protocol in terms of timing between doses BCG: Bacillus of Calmette-Guerin

Dose Phase	Interval between doses			
	On-Time (≤14 days)	Delayed (15-28)	Late (>28)	Data unavailable
BCG Incomplete (N=24)	16 (66%)	2 (8%)	1 (4%)	5
Maintenance completed (N=14)	10 (72%)	2 (14%)	0 (0%)	2

Tumor characteristics showed a high prevalence of high-grade tumors in both non-completers and completers, as detailed in Tables 3, 4.

**Table 3:** Tumor characteristics of patients did not complete BCG CIS: Carcinoma in Situ BCG: Bacillus Calmette Guérin

Initial stage	Ta	13 (54.2%)
	T1	9 (37.5%)
	CIS	1 (4.2%)
Grade (Low/High)	Low	3 (12.5%)
	High	13 (54.2%)
Concomitant CIS (Yes/No)	Yes	7 (29.2%)
	No	14 (58.3%)
	CIS only finding present	1 (4.2%)
Tumour size	<2.5 cm	6 (25.0%)
	> 2.5 cm	11(45.8%)
	Size not recorded	6 (25.0%)
Multifocality	Unifocal	13 (54.2%)
	Multifocal	9 (37.5%)
	Focality not recorded	1 (4.2%)

**Table 4:** Tumor characteristics of patients who completed BCG CIS: Carcinoma in Situ BCG: Bacillus Calmette Guérin

Tumour characteristics of patients who completed BCG		
Initial stage	Ta	7 (50.0%)
	T1	3 (21.4%)
Grade (Low/High)	Low	2 (14.3%)
	High	8 (57.1%)
Concomitant CIS (Yes/No)	Yes	5 (35.7%)
	No	5 (35.7%)
Tumour size	<2.5 cm	0 (0%)
	> 2.5 cm	4 (28.6%)
	Size not recorded	6 (42.9%)
Multifocality	Unifocal	4 (28.6%)
	Multifocal	4 (28.6%)
	Focality not recorded	1 (7.1%)

## Discussion

Previous literature reports that 30% to 40% of patients treated with BCG for NMIBC fail their therapy either through recurrence or progression. Local or systemic side-effects of BCG therapy range from mild, tolerable to severe adverse effects leading to stopping treatment. Examples of local side effects are dysuria and cystitis symptoms, haematuria and epididymo-orchitis, and fever and malaise as systemic side effects. BCG therapy completion is associated with better survival rates compared to patients who fail to complete therapy [5,6]. 

Aksakalli T et al. reported that 8.3% of patients in their study developed BCG-related side effects, which can endanger their lives. Clinicians should consider risk factors, such as tumor size and the presence of CIS, to minimize the frequency of side effects. Dose reduction and adding quinolone prophylactically may help mitigate BCG-related adverse effects [6]. In the present cohort, 60% of patients did not complete the prescribed course. However, only 18% discontinued because of intolerance or significant toxicity; most non-completions were due to logistical barriers and patient decisions, suggesting that logistical or undocumented patient-choice factors were the dominant drivers of non-completion.

Ferro M et al. found that approximately 50% of bladder cancer cases are connected to smoking, making it the most important risk factor. Tumor characteristics, including high-grade T1, lymphovascular invasion, and multifocality, directly influence in a negative manner the success of BCG treatment. Providing an induction course of BCG after TURB for two to six weeks, followed by a full course of maintenance for one to three years, decreases tumor recurrence and progression [7,8]. Furthermore, Moon YJ et al. reported that during the COVID-19 pandemic, a one-year BCG maintenance course was recommended in high-risk NMIBC patients [9]. In our study, the smoking status was documented in only 25% of the patients (n=10), which precluded meaningful statistical analysis of its association with treatment completion. This limitation underscores the need for comprehensive documentation in future studies to explore potential correlations between smoking status and BCG adherence. In our cohort, the distribution of high-grade tumors and multifocality was similar between patients who completed BCG therapy and those who did not, which suggests that tumor characteristics were not significant determinants of BCG completion in this population. That non-completion was more likely driven by non-clinical factors such as logistical barriers and patient choice. Although progression, cystectomy, and survival were collected, these outcomes were not reported due to limited sample size and short follow-up, which precluded meaningful interpretation. This decision aimed to avoid introducing bias through incomplete or non-generalizable results.

The compliance status with guidelines for NMIBC includes various factors, including educational, logistical, financial, social, psychological, and environmental influences. A summary of this compliance can provide insights into the diagnosis, treatment, and follow-up practices in daily clinical settings. Analyzing this data can support the development of strategies aimed at improving adherence to established guidelines [10]. In our study, a significant portion of patients, specifically 66%, who did not complete their BCG treatment received their therapy with an interval of less than two weeks between doses. In contrast, adherence improved to 72% when evaluating those who received therapy within this two-week window. Additionally, 14% of patients had their treatments scheduled within a four-week interval, while there were no documented instances of therapy being delayed beyond one month. Moreover, it has been observed that certain factors positively correlate with better compliance. Specifically, being affiliated with well-regarded teaching hospitals is linked to higher adherence to these guidelines [11].

This study has several limitations. As a retrospective, single-center analysis with a small study group (n=40), it is susceptible to selection bias and limited generalizability. Additionally, incomplete documentation of key clinical variables, such as smoking status, tumor size, and focality, further restricts the ability to identify statistically significant relationships or draw robust conclusions. These limitations reflect the challenges of real-world clinical documentation and underscore the need for prospective, multicenter studies with standardized data collection to better identify modifiable risk factors for BCG failure.

## Conclusions

In this cohort of 40 high-risk NMIBC patients treated with BCG, 60% did not complete the full maintenance schedule. Toxicity accounted for only a minority of discontinuations; most appear related to logistical or undocumented reasons. Documentation of smoking history and tumor measurements was frequently incomplete despite their prognostic importance.

To improve outcomes, structured patient support programmes, improved documentation standards, and proactive toxicity management strategies (e.g., dose adjustment, prophylactic antibiotics) should be implemented. Larger, prospectively collected datasets are required to identify modifiable risk factors for BCG failure in our population.
